# Epidemiological characteristics of Mycoplasma pneumoniae in hospitalized children before during and after the COVID-19 pandemic in xi’an China

**DOI:** 10.1038/s41598-026-38940-7

**Published:** 2026-02-06

**Authors:** Na Liu, Yang Wang, Tao-Min Bai, Fang-Fang Ma, Tian-Tian Han, Huo-Lan Zhu, Xiao-Yan Zhang

**Affiliations:** 1 Department of Pediatrics, Na Liu Master, Shaanxi Provincial People’s Hospital, Xi’an, 710068 Shaanxi China; 2 Department of Pediatrics, Yang Wang Master, Shaanxi Provincial People’s Hospital, Xi’an, 710068 Shaanxi China; 3 Department of Pediatrics, Tao-Min Bai Master, Shaanxi Provincial People’s Hospital, Xi’an, 710068 Shaanxi China; 4 Department of Pediatrics, Fang-Fang Ma Master, Shaanxi Provincial People’s Hospital, Xi’an, 710068 Shaanxi China; 5 Department of Pediatrics, Tian-Tian Han Master, Shaanxi Provincial People’s Hospital, Xi’an, 710068 Shaanxi China; 6 Shaanxi Provincial Clinical Research Center for Geriatric Medicine, Shaanxi Provincial People’s Hospital, Xi’an, 710068 China; 7 Department of Pediatrics, Shaanxi Provincial People’s Hospital, Xi’an, 710068 Shaanxi China

**Keywords:** COVID-19, Epidemiology, Mycoplasma pneumoniae, Pediatric infection, Medical research, Paediatric research, Risk factors

## Abstract

**Background:**

Mycoplasma pneumoniae (MP) is a leading cause of community-acquired pneumonia in children. The COVID-19 pandemic and associated public health interventions have significantly influenced the epidemiology of respiratory infections. This single-center retrospective cohort study aimed to evaluate changes in MP infection patterns among children before, during, and after the COVID-19 pandemic.

**Methods:**

We analyzed data from 15,718 pediatric patients (aged 1–18 years) with CAP admitted to Shaanxi Provincial People’s Hospital between January 2017 and December 2023. The study periods were defined as pre-pandemic (January 1, 2017, to January 22, 2020), pandemic (January 23, 2020, to December 11, 2022), and post-pandemic (December 12, 2022, to December 31, 2023). Epidemiological characteristics of MP infections were assessed using descriptive statistics and regression analysis.

**Results:**

Among the 15,718 patients, 5,454 (34.7%) tested positive for MP. The highest positivity rate was observed in children aged > 6 years (52.4%), with a male predominance across all age groups. Most infections occurred in autumn (41.5%). The MP positivity rate was lowest during the pandemic period and highest in the post-pandemic period (*P* < 0.001). Regression analysis indicated a broadening of the susceptible age range following the pandemic onset.

**Conclusion:**

COVID-19 containment measures altered the transmission dynamics of MP, affecting demographic characteristics such as age distribution and seasonal trends. Continuous surveillance of MP epidemiology in the post-pandemic era is recommended to inform clinical management and public health strategies.

Huo-Lan Zhu, doctor associate chief physician, Shaanxi Provincial Clinical Research Center for Geriatric Medicine, Shaanxi Provincial People’s Hospital, Xi’an 710068, China.telephone:02985251331, E-mail: huolanzhu@spph-sx.ac.cn.

Xiao-Yan Zhang, Master, doctor associate chief physician, Department of Pediatrics, Shaanxi Provincial People’s Hospital, Xi’an 710068, Shaanxi, China.elephone:02985251331, E-mail: ek3245@126.com.

## Introduction

*Mycoplasma pneumoniae* (MP) is a common pathogen that causes community-acquired pneumonia (CAP) globally^1^. Mycoplasma pneumoniae pneumonia (MPP) is responsible for 10–40% of pneumonia-related hospitalizations in children^2,3^. It is particularly dangerous in school-aged children and adolescents, who have high incidence and mortality rates^4^. In most cases, MP infections resolve independently. However, they can lead to persistent pneumonia and injuries outside the lungs, resulting in severe complications and even death^5^. Therefore, children are at higher risk of developing MP infections and have high incidence and mortality rates^6^.

MP is self-replicating prokaryotic microorganisms that are primarily transmitted through respiratory droplets and close contact^7,8^. It is atypical compared with other pathogens for multiple reasons^9^. It is one of the smallest self-replicating organisms. It possesses a reduced and stable genome (0.8 Mbp), lacks a cell wall, grows slowly (with a generation time of 6 h), and requires close contact for transmission^10^. Furthermore, it induces atypical pneumonia, which may involve host-cell-mediated immunity^11^. The incubation period spans one–three weeks, with individuals capable of remaining contagious from the onset of symptoms to multiple weeks after recovery^12^.

MP infections can occur during any season, with peaks occurring every few years^13^. These infections are more frequently observed in autumn and winter in northern China and more widespread in summer and autumn in southern China^12^. Since the emergence of the coronavirus disease (COVID-19) in December 2019, non-pharmaceutical interventions (NPIs) such as wearing masks, hand hygiene, and social distancing have been implemented to control the spread of SARS-CoV-2^6^. Although these measures efficiently decreased transmission of SARS-CoV-2, they also considerably reduced MP detection rates, essentially disrupting the previously established epidemiological trends^5^. Similar to other respiratory pathogens, the incidence of MP infections in children significantly decreased immediately following the introduction of NPIs^9^. However, with the dissolution of pandemic restrictions, the incidence of MP infections has remarkably increased, garnering widespread attention and warranting further investigation^14^.

However, information on the long-term epidemiological characteristics of MP infections during the various stages of the COVID-19 pandemic in China remains scarce. However, information on the long-term epidemiological characteristics of MP infections during the various stages of the COVID-19 pandemic in China remains scarce. While recent studies from regions such as Chongqing have reported data extending into late 2023, comprehensive analysis capturing the full post-pandemic calendar year remains limited^6,14–16^. Furthermore, data from Northwestern China-a region with distinct climatic and demographic characteristics-are still markedly underrepresented. To address these gaps, this retrospective study conducted a seven-year (2017–2023) single-center analysis in Xi’an. This timeframe uniquely encompasses the entirety of 2023, allowing for a complete assessment of the infection surge throughout the first full post-pandemic year. Moreover, data from Xi’an provide crucial insights into regional variations in MP transmission.

## Methods

### Study design and subjects

This retrospective cohort study included pediatric patients (aged 1–18 years) diagnosed with community-acquired pneumonia (CAP) who underwent Mycoplasma pneumoniae (MP) testing at Shaanxi Provincial People’s Hospital between January 1, 2017, and December 31, 2023. All pediatric patients admitted with a diagnosis of CAP during the study period were routinely tested for MP as part of standard clinical care. Demographic, clinical, and laboratory data were extracted from the hospital’s Big Data Engineering Center.

## Inclusion and exclusion criteria

Patients were included in the study if they met the following criteria: (1) Hospital admission diagnosis of community-acquired pneumonia (CAP)^17,18^;and (2) Laboratory-confirmed positive infection with Mycoplasma pneumoniae according to the study definition (see below). Patients with severe underlying conditions (including congenital heart disease, primary immunodeficiency, chronic lung disease, malignancy, or undergoing immunosuppressive therapy) were excluded.All community-acquired pneumonia diagnoses in pediatric patients were confirmed by imaging studies. This study primarily relied on chest X-ray findings.

## MPP diagnostic criteria

Throughout the study period (2017–2023), Mycoplasma pneumoniae pneumonia (MPP) diagnosis was based on serological test results, reflecting standard clinical practice at our center. Inpatients with community-acquired pneumonia (CAP) were diagnosed with MPP if they met any of the following serological criteria, consistent with national guidelines (Zhu Futang’s Practical Pediatrics, 8th Edition; Guidelines for the Diagnosis and Treatment of Community-Acquired Pneumonia in Children (2015)):(1)A single serum particle agglutination (PA) antibody titer ≥ 1:160;(2) A positive MP-IgM antibody test result.In the data analysis, we identified some patients who met both positive criteria simultaneously. To avoid double counting and ensure case independence, each patient was included only once in the final cohort and assigned to a single diagnostic category. Therefore, the case counts reported for each detection method (MP-IgM and PA) in this paper represent independent counts.Although nucleic acid testing (PCR) for MP was introduced in late 2023, serological criteria were uniformly applied throughout the retrospective cohort to ensure diagnostic consistency.For the purposes of this study, and in line with the diagnostic criteria outlined above, patients meeting these serological criteria are referred to as having MP-positive CAP or as hospitalized children with MP infection.

## Study periods and subgroup analysis

The research phase and subgroup analysis phase are divided into three stages based on key adjustments to China’s national COVID-19 prevention and control policies, consistent with recent epidemiological findings on MP in China^6^:(1)Phase I(Pre-pandemic phase): January 1, 2017, to January 22, 2020 (ending on the eve of Wuhan’s lockdown, ),(2)Phase II༈Pandemic Phase༉: January 23, 2020, to December 11, 2022 (encompassing the period of strict “dynamic zero-COVID” policy implementation until the eve of the national announcement of the “New Ten Guidelines”),(3)Phase III༈Post-pandemic phase༉: December 12, 2022, to December 31, 2023 (commencing with the implementation of the “New Ten Guidelines,” marking the end of extensive non-pharmaceutical interventions).Patients were further categorized by age (1–3 years, 3–6 years, > 6 years) and season of admission: spring (March–May), summer (June–August), autumn (September–November), and winter (December–February).

## Ethics approval

This study was approved by Medical Research Ethics Committee of Shaanxi Provincial People’s Hospital.All procedures were performed in accordance with the ethical standards of the institutional committee and with the 1964 Helsinki declaration and its later amendments.

### Statistical analysis

Categorical variables were presented as numbers (n) and percentages (%), with 95% confidence intervals (CI) calculated for proportions. Comparisons between groups were performed using the Chi-square test (χ² test). Continuous variables that did not follow a normal distribution were expressed as median (M) and interquartile range (IQR), and comparisons between groups were conducted using the Kruskal-Wallis rank-sum test. We used multivariable logistic regression to evaluate independent risk factors (including age, gender, season, and pandemic phase) for MP positivity. Furthermore, using the restricted cubic spline regression model in R software (version 4.4.2), nonlinear associations between age and MP-positive detection rate at different stages of the pandemic were analyzed. A two-sided P-value of < 0.05 was considered statistically significant. All statistical analyses were performed using R software (version 4.4.2).

## Results

### Positive detection rates of MP across age groups

This comprehensive analysis included 15,718 children aged 1–18 years (Table 1). The children were divided into three age groups: 7,761 infants (1–3 years), 4,737 preschool children (3–6 years), and 3,220 school-aged children and adolescents (> 6 years). A total of 5,454 MP-positive patients were confirmed based on serological criteria.Based on the specific diagnostic testing methods used in each phase, the distribution of these cases is as follows: In Phase I (pre-pandemic), 1,299 and 1,656 cases were confirmed by positive MP-IgM antibody testing and serum particle agglutination (PA) antibody titers ≥ 1:160, respectively. During Phase II (pandemic), the corresponding figures were 319 cases (MP-IgM) and 583 cases (PA titer). In Phase III (post-pandemic), 1,166 cases were confirmed via MP-IgM testing and 1,726 cases via PA titer testing. As described in the Methods section, to ensure consistency throughout the study period, nucleic acid testing (PCR) was not used as an initial case identification method.

**Table 1:** Positive detection rates and 95% confidence intervals of MP infection across age groups.

Variables	All patients	1–3 years	3–6 years	> 6 years
Total Number	15,718	7761	4737	3220
MP positive number	5454	1957	1811	1686
Positive rate, % (95%CI)	34.7 (33.9–35.5)	25.2 (24.2–26.2)	38.2 (36.8–39.6)	52.4 (50.7–54.1)
Gender n % (95%CI)				
Female	2643 (31.3, 30.2–32.4)	956 (22.2, 21.0-23.5)	821 (33.6, 31.8–35.4)	866 (51.6, 49.4–53.8)
Male	2811 (38.6, 37.5–39.7)	1001 (29.0, 27.6–30.4)	990 (43.1, 41.2–45.0)	820 (53.1, 50.9–55.3)
*P* Value	< 0.001	< 0.001	< 0.001	0.413
Season n % (95%CI)				
Spring	1082 (28.1, 26.6–29.6)	500 (23.3, 21.5–25.1)	388 (32.2, 29.6–34.8)	194 (39.3, 35.3–43.3)
Summer	975 (32.6, 30.9–34.3)	407 (26.6, 24.3–28.9)	317 (34.0, 31.0–37.0)	251 (47.3, 43.4–51.2)
Autumn	2114 (41.5, 40.1–42.9)	583 (26.6, 24.7–28.5)	681 (44.4, 41.9–46.9)	850 (62.0, 59.6–64.4)
Winter	1283 (33.9, 32.3–35.5)	467 (24.6, 22.6–26.6)	425 (39.9, 37.1–42.7)	391 (47.5, 44.3–50.7)
*P* Value	< 0.001	0.036	< 0.001	< 0.001
Pandemic phases, n % (95%CI)				
Phase I	2568 (25.9, 25.0-26.8)	1170 (21.4, 20.3–22.5)	886 (30.8, 29.1–32.5)	512 (32.7, 30.4–35.0)
Phase II	751 (30.2, 28.3–32.1)	309 (25.1, 22.7–27.5)	270 (31.4, 28.5–34.3)	172 (43.5, 39.0–48.0)
Phase III	2135 (64.1, 62.7–65.5)	478 (44.5, 41.7–47.3)	655 (65.3, 62.6–68.0)	1002 (79.7, 77.8–81.6)
*P* Value	< 0.001	< 0.001	< 0.001	< 0.001
Length of stay, days, M (IQR)	8 (7, 10)	7 (7, 9)	8 (7, 10)	9 (7, 11)
P Value (Kruskal-Wallis)	< 0.001			

Data are presented as n (%) with 95% CI, unless otherwise specified. Comparisons were made using the Chi-square test. The length of stay is presented as median (IQR), and compared using the Kruskal-Wallis test. MP positive number: number of hospitalized children with a positive test for Mycoplasma pneumoniae.

**Fig. 1:**
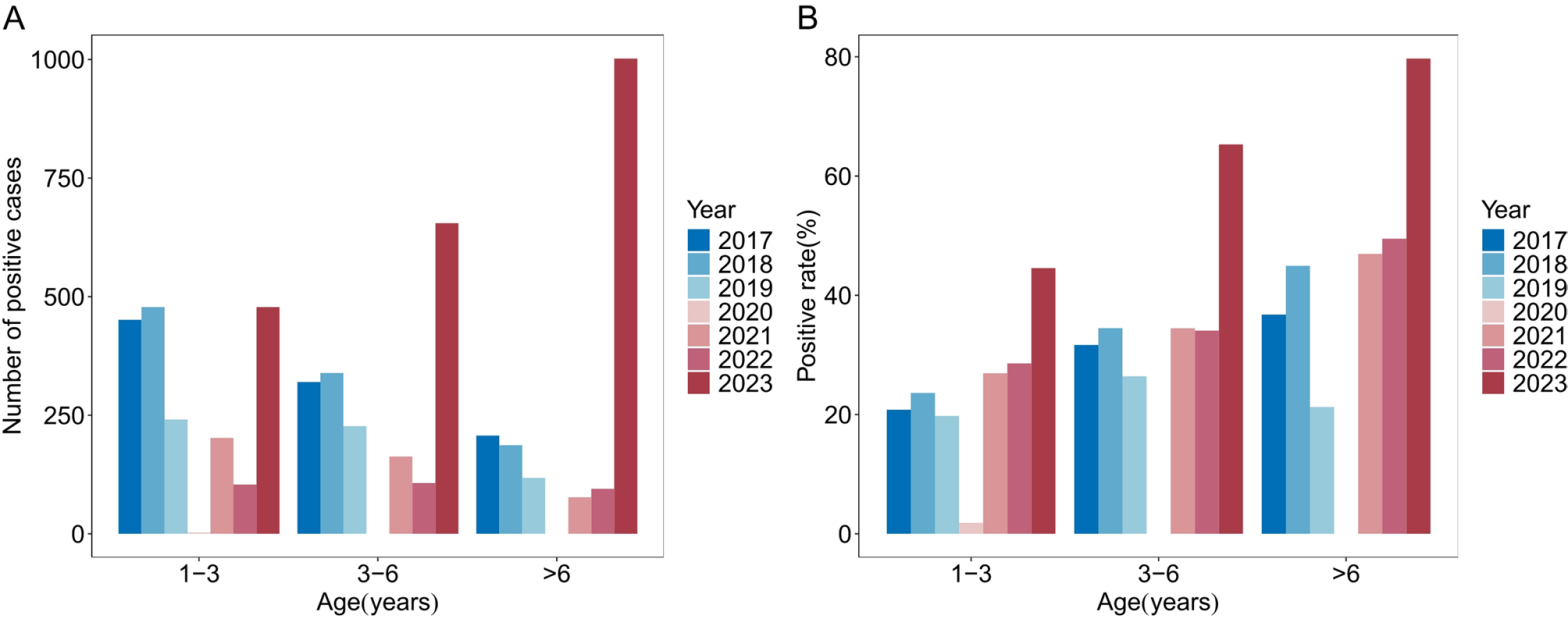
Age distribution of MP infection from 2017 to 2023.(**A**) MP-positive case numbers and (**B**) positive rates at different ages.

**Fig. 2:**
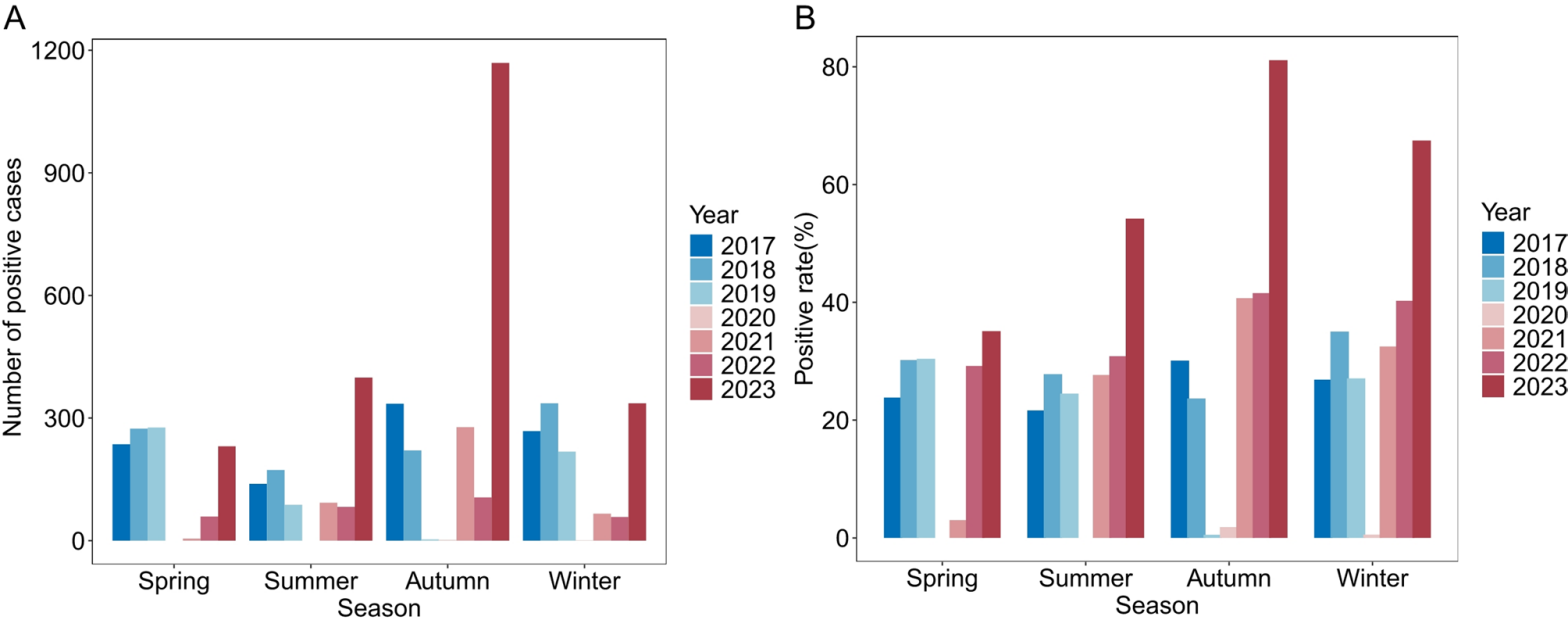
Seasonal distribution of MP infection from 2017 to 2023.(**A**) MP-positive case number and (**B**) positive rate in different seasons.

The lowest positivity rate was observed in patients aged 1–3 years (25.2%), which gradually increased with age and reached its highest positivity rate in patients aged > 6 years (52.4%)(Figs. 1A, B). The incidence was higher in males than in females across all age groups. The positivity rates in male patients aged 1–3 years (29.0%) and in male patients aged 3–6 years (43.1%) were significantly higher (*P* < 0.001). Autumn demonstrated the highest positivity rate for every age group (26.6% for those aged 1–3 years, 44.4% for those aged 3–6 years, and 62.0% for those > 6 years) (Figs. 2A, B). Positivity rates differed significantly across the three phases (*P* < 0.001), being lowest during Phase II (pandemic), intermediate in Phase I (pre-pandemic), and highest in Phase III (post-pandemic). The median length of hospital stay increased with age, from 7 days (IQR: 7–9) in the 1–3 years group to 9 days (IQR: 7–11) in the > 6 years group (*P* < 0.001).

### Demographic and clinical characteristics of MP-Positive patients across pandemic phases

We analyzed 5,454 MP-positive cases (Table 2), distributed as 2,568 in Phase I (pre-pandemic), 751 in Phase II (pandemic), and 2,135 in Phase III (post-pandemic). The demographic composition of MP-positive cases shifted significantly across the pandemic phases (*P* < 0.001). Notably, the proportion of cases in the 1–3 years age group decreased markedly, from 45.6% (95% CI: 43.6–47.5) in Phase I to 22.4% (20.6–24.2) in Phase III. Conversely, the proportion in the > 6 years group increased substantially from 19.9% (18.4–21.5) to 46.9% (44.8–49.1), making it the predominant group post-pandemic. A borderline significant difference in gender distribution was also noted across the phases (*P* = 0.048). Seasonality was markedly altered (*P* < 0.001), with a pronounced autumn peak emerging in later phases (21.8% in Phase I vs. 54.8% in Phase III). Clinical metrics intensified in Phase III: the median hospital stay increased from 7 days (IQR: 7–10) to 9 days (IQR: 7–10), and the median total hospitalization cost rose from 4,948 CNY (IQR: 4,066–6,433) to 6,390 CNY (IQR: 5,063–8,002) (both *P* < 0.001).

**Table 2:** Demographic and clinical characteristics of MP-positive cases before, during, and after the COVID-19 pandemic.

Characteristic	Overall *N* = 5,454	Pandemic phase			*P*-value
		Phase I *N* = 2,568	Phase II *N* = 751	Phase III *N* = 2,135	
Age group, n (%, 95% CI)					< 0.001
1–3	1957 (35.9, 34.6–37.2)	1170 (45.6, 43.6–47.5)	309 (41.1, 37.6–44.7)	478 (22.4, 20.6–24.2)	
3–6	1811 (33.2, 31.9–34.5)	886 (34.5, 32.7–36.3)	270 (36.0, 32.5–39.4)	655 (30.7, 28.7–32.6)	
> 6	1686 (30.9, 29.7–32.1)	512 (19.9, 18.4–21.5)	172 (22.9, 19.9–25.9)	1002(46.9, 44.8–49.1)	
Gender, n (%, 95% CI)					0.048
Female	2643 (48.5, 47.1–49.8)	1211 (47.2, 45.2–49.1)	353 (47.0, 43.4–50.6)	1079(50.5, 48.4–52.7)	
Male	2811 (51.5, 50.2–52.9)	1357(52.8, 50.9–54.8)	398 (53.0, 49.4–56.6)	1056(49.5, 47.3–51.6)	
Season, n (%, 95% CI)					< 0.001
Autumn	2114 (38.8, 37.5–40.1)	559 (21.8, 20.2–23.4)	386 (51.4, 47.8–55.0)	1169 (54.8, 52.7–56.9)	
Spring	1082 (19.8, 18.8–20.8)	787 (30.6, 28.9–32.3)	64 (8.5, 6.5–10.5)	231 (10.8, 9.5–12.1)	
Summer	975 (17.9, 16.9–18.9)	400 (15.6, 14.2–17.0)	176 (23.4, 20.4–26.4)	399 (18.7, 17.0–20.4)	
Winter	1283 (23.5, 22.4–24.6)	822 (32.0, 30.2–33.8)	125 (16.6, 14.0–19.2)	336 (15.7, 14.2–17.2)	
Length of stay, days, Median (IQR)	8 (7, 10)	7 (7, 10)	7 (7, 9)	9 (7, 10)	< 0.001
Total cost, Median (IQR)	5498 (4409, 7198)	4948 (4066, 6433)	5205 (4460, 6370)	6390 (5063, 8002)	< 0.001

Data are presented as n (%, 95% CI) for categorical variables and median (interquartile range) for continuous variables. The Chi-squared test was used for categorical variables, and the Kruskal-Wallis test for continuous variables. P-value < 0.05 was considered statistically significant.

### Evolution of chest imaging findings

Chest imaging manifestations shifted substantially across the pandemic phases (Table 3).Overall, increased pulmonary markings were the most common finding (62.0%), followed by pulmonary consolidation (27.0%).A striking reversal in the prevalence of these two patterns occurred over time. In Phase I, increased pulmonary markings predominated (91.1%), while consolidation was uncommon (3.9%). By Phase III, this pattern had inverted: consolidation became the dominant finding (62.1%), and the prevalence of increased markings declined to 41.6%. The occurrence of focal bronchiolitis decreased from 5.5% to 1.3%. Atelectasis and pleural effusion remained rare (< 1% in all phases).

**Table 3:** Imaging analysis of MP-positive cases before, during, and after the COVID-19 pandemic.

Imaging finding	Overall *N* = 5,454	Pandemic phase		
		Phase I *N* = 2,568	Phase II *N* = 751	Phase III *N* = 2,135
Increased and blurred lung markings, n (%, 95% CI)	3379(62.0, 60.7–63.3)	2340(91.1, 90.0–92.2)	551(73.4, 70.2–76.6)	888 (41.6, 39.5–43.7)
Pulmonary consolidation, n (%, 95% CI)	1472 (27.0, 25.8–28.2)	99 (3.9, 3.1–4.7)	48 (6.4, 4.6–8.2)	1325(62.1, 60.0–64.2)
Atelectasis, n (%, 95% CI)	19 (0.3, 0.2–0.4)	2 (0.1, 0.0–0.2)	2 (0.3, 0.0–0.7)	14 (0.7, 0.3–1.1)
Pleural effusion, n (%, 95% CI)	13 (0.2, 0.1–0.3)	4 (0.2, 0.0–0.4)	3 (0.4, 0.0–0.8)	6 (0.3, 0.1–0.5)
Localized bronchiolitis manifestations, n (%, 95% CI)	199 (3.6, 3.1–4.1)	140 (5.5, 4.6–6.4)	31 (4.1, 2.7–5.5)	28 (1.3, 0.8–1.8)

Data are presented as absolute frequencies with percentages and 95% confidence intervals(95%CI).

### Monthly epidemiological trends from 2017 to 2023

**Fig. 3:**
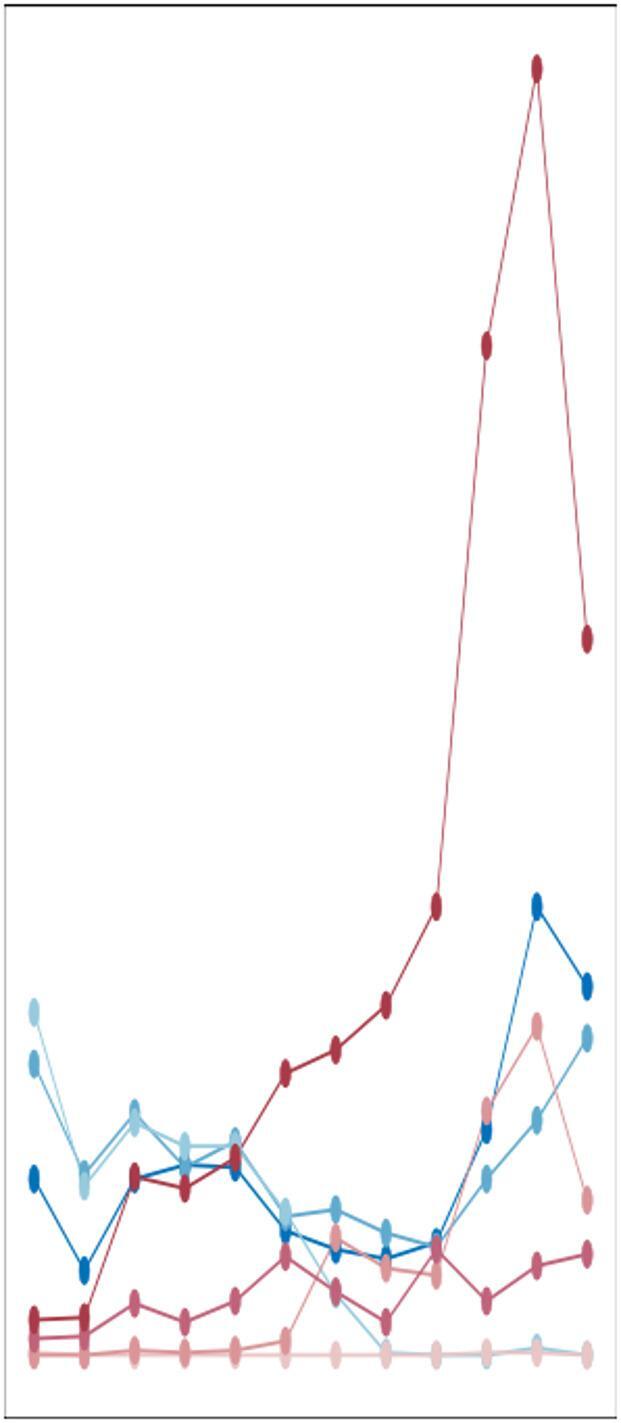
Monthly distribution of MP infection from 2017 to 2023.(**A**) MP-positive numbers and (**B**) positive rates in different months.

Monthly trends in Mycoplasma pneumoniae (MP) positivity exhibited distinct patterns before, during, and after the pandemic period (Figs. 3A, B). Prior to the pandemic (2017–2019), cases typically increased from September, peaking in late autumn or early winter (November–December), followed by a decline.This pattern was profoundly disrupted during the pandemic. In 2020, both case numbers and positivity rates declined significantly and remained low throughout the year. A partial resurgence was observed in 2021, with rates increasing after September and peaking in November, although remaining below pre-pandemic levels.In the post-pandemic year of 2023, the trend before May resembled pre-pandemic patterns. However, an anomalous surge began earlier, with positivity rising from April, accelerating notably by September, and then declining after November. This post-pandemic peak substantially exceeded all pre-pandemic levels.

### Multivariable logistic regression analysis of risk factors for MP positivity

The results of the multivariable logistic regression analysis, adjusted for all covariates, are presented in the Table 4. Age remained a significant independent risk factor, with each additional year of age associated with a 2% increase in the odds of MP infection (adjusted odds ratio [aOR] 1.02, 95% CI 1.02–1.03; *P* < 0.001). Male sex was also independently associated with higher odds compared to female sex (aOR 1.07, 95% CI 1.05–1.08; *P* < 0.001).

**Table 4:** Multivariable logistic regression analysis of factors associated with MP positivity.

Characteristic	Category	aOR	Cl_lower	Cl_upper	*p*-value
Age	Per year	1.02	1.02	1.03	< 0.001
Gender	Male vs. Female	1.07	1.05	1.08	< 0.001
Season	Spring vs. Autumn	0.93	0.92	0.95	< 0.001
Season	Summer vs. Autumn	0.94	0.92	0.96	< 0.001
Season	Winter vs. Autumn	0.99	0.97	1.01	0.3
Pandemic	Phase 2 vs. Phase 1	1.03	1.01	1.05	0.003
Pandemic	Phase 3 vs. Phase 1	1.40	1.38	1.43	< 0.001

aOR, adjusted odds ratio; CI, confidence interval. Reference categories: Gender, Female; Season, Autumn; Pandemic phase, Phase I. The model was adjusted for all variables listed in the table.

A strong seasonal pattern was observed, with autumn conferring the highest risk. Compared to autumn, the odds of infection were significantly lower in both spring (aOR 0.93, 95% CI 0.92–0.95; *P* < 0.001) and summer (aOR 0.94, 95% CI 0.92–0.96; *P* < 0.001). The odds in winter did not differ significantly from those in autumn (aOR 0.99, 95% CI 0.97–1.01; *P* = 0.3).

Furthermore, the pandemic phase was a strong independent predictor of MP infection. Relative to Phase I, the odds of infection were significantly higher in Phase II (aOR 1.03, 95% CI 1.01–1.05; *P* = 0.003) and substantially higher in Phase III (aOR 1.40, 95% CI 1.38–1.43; *P* < 0.001).

### Nonlinear association between age and MP positivity

**Fig. 4:**
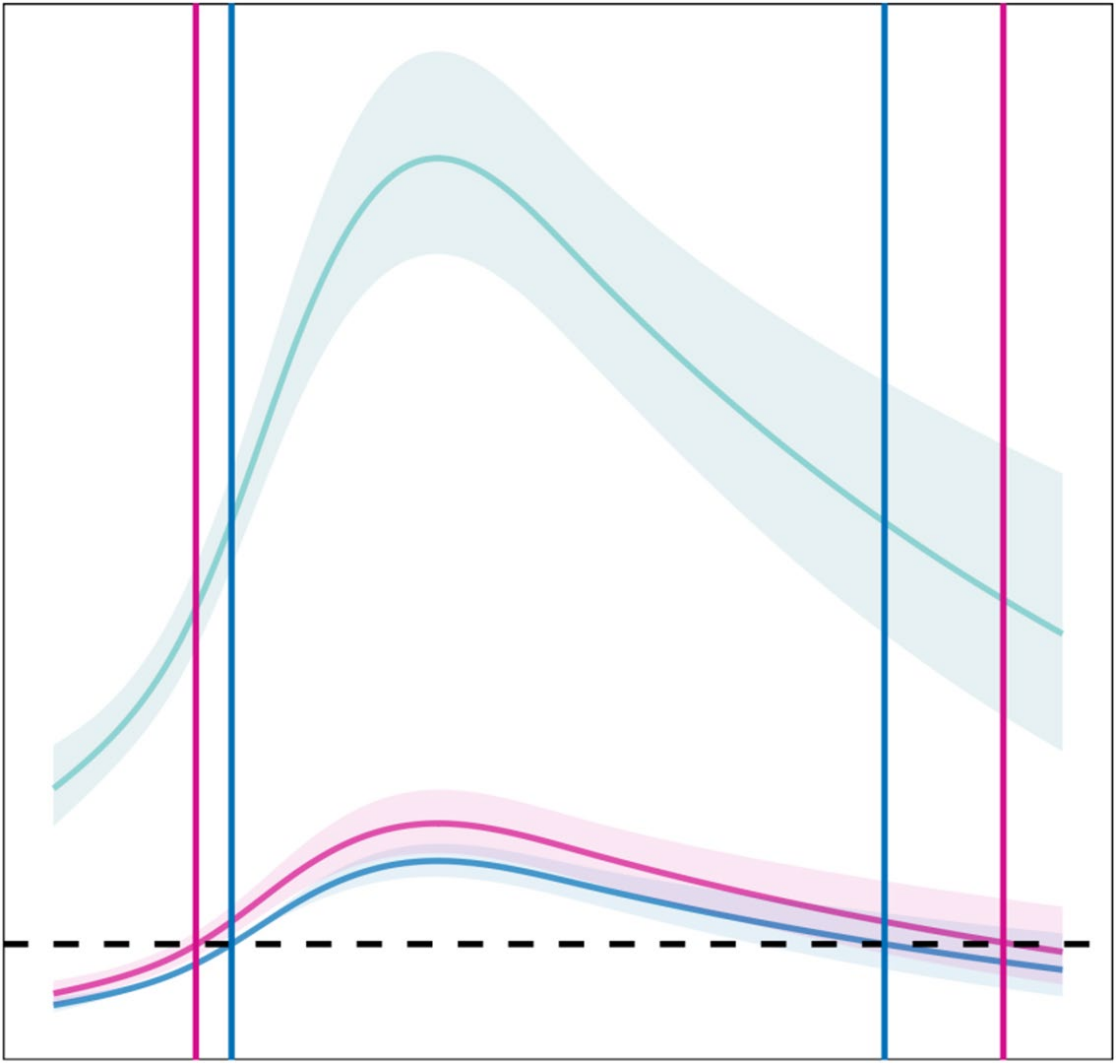
Nonlinear association between age and risk of MP infection estimated. using a restricted cubic spline regression model.

A estricted cubic spline regression model was employed to examine the nonlinear relationship between age and the risk of MP infection across the three pandemic phases. The analysis revealed three key findings. First, the risk of MP infection increased progressively with age during all phases, reaching its highest level in Phase III (post-pandemic). Second, the age range of susceptible children broadened, extending from 4 to 15 years in the pre-pandemic phase to 3.1–17 years during the pandemic. Finally, following the pandemic, the incidence of MP infection increased across all age groups.(Fig. 4 ).

## Discussion

Following the COVID-19 outbreak, stringent non-pharmaceutical interventions (NPIs) implemented in Xi’an, China, until December 2022 were associated with a significant reduction in Mycoplasma pneumoniae (MP) transmission, as evidenced by the markedly lower MP positivity rates among hospitalized children with community-acquired pneumonia (CAP) during the pandemic compared to the pre-pandemic period.This aligns with known respiratory droplet transmission dynamics of MP and findings from other regions^9,19,20^. The subsequent resurgence of MP infections following the lifting of restrictions is consistent with the concept of immune debt^9^,wherein reduced pathogen exposure during NPIs leads to a larger susceptible population. Alternative explanations, such as delayed healthcare-seeking during lockdowns and heightened clinical vigilance post-pandemic, may have also contributed to the observed increase in detection rates^21–24^.

Our analysis identified the pandemic phase as a strong independent predictor of testing positive for MP. The substantial increase in odds in Phase III highlights the profound impact of NPI lifting. The slight but significant increase in odds during Phase II (amidst NPIs) may reflect a shift in healthcare-seeking behavior, leading to a higher proportion of testing among more symptomatic cases, thus elevating the observed positivity rate despite potentially low community transmission^25^.

Age was a continuous risk factor, with each additional year associated with increased odds of MP positivity. This finding, supported by our restricted cubic spline analysis showing a broadened susceptible age range (3.1–17 years) post-pandemic, underscores the high disease burden in school-aged children and adolescents, likely due to their dense social contacts^9^.The consistent, though modest, increased risk associated with male sex across all age groups warrants further investigation into its biological or behavioral basis.

The epidemiological pattern of MP was also characterized by an evolving seasonality. Our model identified autumn as the season of highest risk post-pandemic, a finding corroborated by our time-series data showing an intense autumnal peak in Phase III that exceeded pre-pandemic levels. This shift suggests that NPIs may have disrupted the endemic circulation of MP, leading to a recalibration of its seasonal dynamics upon re-emergence^12,26^.

In the post-pandemic phase, children with MP-positive CAP experienced prolonged hospital stays and incurred higher costs. These findings may indicate increased healthcare resource utilization and potentially reflect heightened disease severity^27^. This interpretation is supported by a significant shift in radiographic patterns, from a pre-pandemic predominance of increased pulmonary markings (91.1%) to a post-pandemic predominance of pulmonary consolidation (62.1%). Pulmonary consolidation is a recognized marker of severe pulmonary inflammation and is itself an independent risk factor for complications in pediatric MPP^28^. However, it is important to consider that changes in clinical imaging indications, modalities (e.g., increased CT utilization), or interpretation practices over time could have contributed to this observed shift, independent of true pathological changes.

Taken together, and despite the need for cautious interpretation of imaging shifts, the observed increases in hospitalization duration, cost, and radiographic consolidation suggest a rise in the clinical severity of post-pandemic MP infections. This potential increase in severity may be attributed to several interrelated mechanisms:1) Immunological debt, leading to more naive older infections and potentially stronger inflammatory responses^9^; 2) Viral co-infections, which we could not systematically assess but are known to complicate clinical presentations^29^; and 3) The potential spread of macrolide-resistant M. pneumoniae (MRMP) strains, which is associated with treatment failure, more severe manifestations, and prolonged hospitalization^30–32^. It is critical to note that the latter remains a hypothesis, as our study lacks molecular data to confirm the prevalence of MRMP or its direct contribution to the outcomes observed.

A crucial limitation of our study is inherent to its hospital-based surveillance design, which precludes direct inference about community transmission dynamics or pathogen virulence^33^. The observed epidemiological and clinical trends could have been confounded by shifts in hospital admission thresholds, healthcare-seeking behavior, and testing practices across the pandemic phases. For instance, a post-pandemic lowering of the threshold for hospitalizing children with respiratory symptoms could have artifactually influenced key metrics such as length of stay and the test positivity rate.

Limitations.

This study has limitations that should be considered when interpreting the findings. First, regarding case definition and selection bias, our diagnosis relied on a single high-titer MP antibody test, which, while clinically practical, cannot definitively distinguish acute infection from carriage or past exposure. Consequently, our cohort is best described as children with MP-positive CAP rather than etiologically confirmed MPP. Furthermore, as a hospital-based study, changes in admission thresholds, healthcare-seeking behaviors, and referral patterns before and after the pandemic may have introduced selection bias, affecting the observed trends in positivity rates and patient characteristics.

Second, key mechanistic data are absent. We did not perform systematic multi-pathogen screening, preventing assessment of co-infections’ role in severity. Critically, we lack molecular data on macrolide resistance, making the discussion about MRMP’s contribution speculative.

Third, our severity assessment has constraints. We used surrogate markers (length of stay, cost, radiographic consolidation) as objective clinical severity data (e.g., oxygen supplementation, ICU admission) were not routinely available. While these surrogates reflect healthcare burden, they are influenced by non-clinical factors like hospitalization policies. Additionally, radiographic interpretations may have been affected by evolving imaging protocols and indications over time.

Despite these limitations, the coherent trend of deterioration across multiple independent dimensions—increased radiographic consolidation, prolonged hospitalization, and higher costs—provides a robust, multi-faceted signal suggesting a genuine post-pandemic shift towards greater clinical burden, unlikely to be explained by any single confounding factor alone. This real-world evidence offers valuable insights for clinical preparedness.

## Conclusion

In conclusion, NPIs significantly altered the epidemiology of MP-positive CAP in children. Post-pandemic trends suggest a shift in age distribution, intensified seasonality, and potentially increased clinical severity and healthcare resource utilization. Sustained surveillance, coupled with future research incorporating molecular resistance testing and multi-pathogen screening, is vital to confirm these trends, elucidate underlying mechanisms, and guide effective clinical and public health responses in the post-pandemic era.

## Data Availability

All data supporting the findings of this study are available in the article and are available from the corresponding author.
